# Benthic meiofaunal community response to the cascading effects of herbivory within an algal halo system of the Great Barrier Reef

**DOI:** 10.1371/journal.pone.0193932

**Published:** 2018-03-07

**Authors:** Quinn R. Ollivier, Edward Hammill, David J. Booth, Elizabeth M. P. Madin, Charles Hinchliffe, Alastair R. Harborne, Catherine E. Lovelock, Peter I. Macreadie, Trisha B. Atwood

**Affiliations:** 1 Centre for Integrative Ecology, School of Life and Environmental Sciences, Faculty of Science Engineering and Built Environment, Deakin University, Melbourne, VIC, Australia; 2 School of Life Sciences, University of Technology Sydney, Sydney, NSW, Australia; 3 Department of Watershed Sciences and Ecology Center, Utah State University, Logan, UT, United States of America; 4 Department of Biological Sciences, Macquarie University, Sydney, NSW, Australia; 5 Hawai’i Institute of Marine Biology, University of Hawai’i, Kane’ohe, HI, United States of America; 6 School of Biological, Earth and Environmental Sciences, University of New South Wales, Sydney, NSW, Australia; 7 Marine Spatial Ecology Laboratory and Australian Research Council Centre of Excellence for Coral Reef Studies, School of Biological Sciences, University of Queensland, Brisbane, QLD, Australia; 8 Department of Biological Sciences, Florida International University, North Miami, Florida, United States of America; 9 Global Change Institute, University of Queensland, St. Lucia, QLD, Australia; 10 School of Biological Sciences University of Queensland, St. Lucia, QLD, Australia; Department of Agriculture and Water Resources, AUSTRALIA

## Abstract

Benthic fauna play a crucial role in organic matter decomposition and nutrient cycling at the sediment-water boundary in aquatic ecosystems. In terrestrial systems, grazing herbivores have been shown to influence below-ground communities through alterations to plant distribution and composition, however whether similar cascading effects occur in aquatic systems is unknown. Here, we assess the relationship between benthic invertebrates and above-ground fish grazing across the ‘grazing halos’ of Heron Island lagoon, Australia. Grazing halos, which occur around patch reefs globally, are caused by removal of seagrass or benthic macroalgae by herbivorous fish that results in distinct bands of unvegetated sediments surrounding patch reefs. We found that benthic algal canopy height significantly increased with distance from patch reef, and that algal canopy height was positively correlated with the abundances of only one invertebrate taxon (Nematoda). Both sediment carbon to nitrogen ratios (C:N) and mean sediment particle size (μm) demonstrated a positive correlation with Nematoda and Arthropoda (predominantly copepod) abundances, respectively. These positive correlations indicate that environmental conditions are a major contributor to benthic invertebrate community distribution, acting on benthic communities in conjunction with the cascading effects of above-ground algal grazing. These results suggest that benthic communities, and the ecosystem functions they perform in this system, may be less responsive to changes in above-ground herbivorous processes than those previously studied in terrestrial systems. Understanding how above-ground organisms, and processes, affect their benthic invertebrate counterparts can shed light on how changes in aquatic communities may affect ecosystem function in previously unknown ways.

## Introduction

Above-ground ecosystem components and their benthic or below-ground counterparts are often studied in isolation of one another. However, the biota in these components are intricately connected through a series of complex interactions and processes [1–3]. For example, it is well known that soil and sediment communities are responsive to changes in the quality and quantity of organic matter entering their food web. These responses can manifest as shifts in the soil or sediment community composition, as well as alter the rate at which these communities perform essential ecosystem processes (e.g., decomposition) [4,5]. Conversely, soil and sediment communities break down dead organic matter, releasing the essential nutrients that fuel plant growth and influence plant diversity [6–8]. Thus, studies that explore the linkages between above-ground and benthic or epibenthic processes are essential for understanding the mechanisms that drive community and ecosystem dynamics.

In terrestrial ecosystems, the cascading effects of herbivore activities on below-ground community composition, transmitted through alterations to plant abundance and/or distributions, have been well documented [2,3,9,10]. These studies demonstrate that above-ground herbivores can alter the community composition of infaunal biota by changing the quantity and/or quality of resource inputs to soil communities, also likely influencing soil abiotic factors such as temperature and moisture [1]. These studies have also shown that herbivore-mediated changes to infauna have implications for nutrient cycling and other ecosystem functions that lead to feedbacks in the above-ground communities, highlighting some of the effects that anthropogenic changes to plant-herbivore systems (e.g., through invasive species or species extirpation) may have on the structure and functioning of terrestrial ecosystems. In contrast, investigations of trophic cascades in aquatic ecosystems have so far focused on infaunal herbivorous consumption of above- and below-ground biomass (e.g. crabs in tidal marshes) [11,12] or predator-prey interactions, and their indirect effects on primary producers [13–15,11] and nutrient cycling [16], while little is currently known regarding the potential indirect effects of herbivory on benthic invertebrate communities [17].

**Fig 1:**
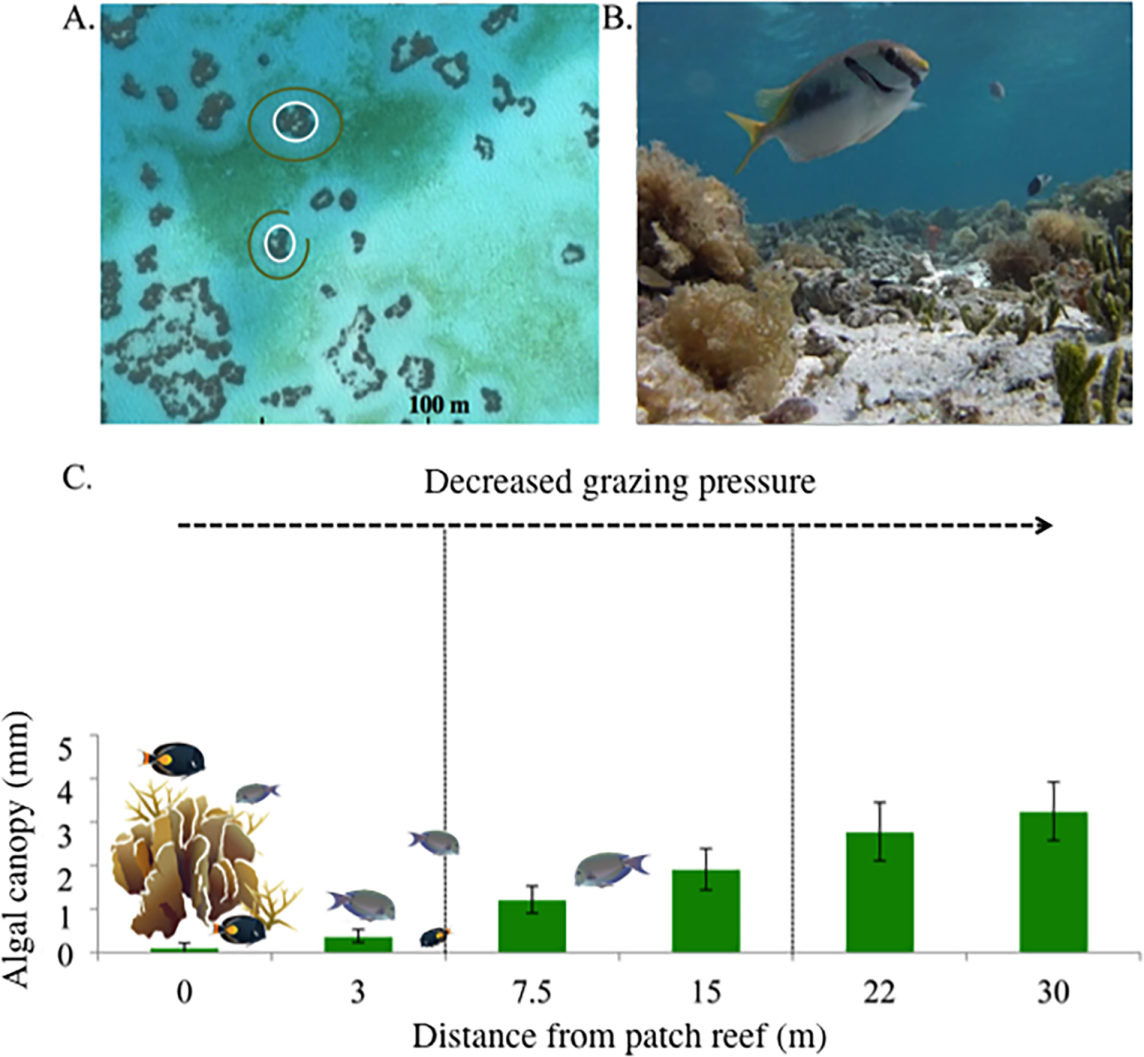
Effects of grazing on benthic algal distribution in a coral reef lagoon ecosystem. A) Satellite image of algal halos within Heron Island lagoon. White lines indicate coral patch reefs; green lines indicate the approximate outer extent of bare sandy substrate (i.e., halos). B) *Siganus doliatus*, a small reef dwelling herbivore taking shelter in a tropical coral patch reef, at least partly as an anti-predator response. Although this particular species likely contributes little to halo formation beyond the reef due to its territorial ‘farming’ habits on the reef itself, the anti-predator sheltering behaviour it displays here is indicative of reef fishes in general. Predation risk is commonly cited as the ultimate mechanism leading to the spatially-constrained herbivore grazing patterns around coral patch reefs that lead to halo formation (photo: A. Harborne). C) Conceptual diagram illustrating how herbivore grazing patterns influence mean algal density and canopy height.

One potential example of cascading effects being transmitted through grazing herbivores in aquatic ecosystems is that of marine ‘grazing halos’. Grazing halos, which occur around patch reefs globally, are caused by the removal of seagrass or macroalgae that results in a distinct band of unvegetated sediment surrounding a patch reef [18–25]. Although the mechanisms behind the development of grazing halos are not fully understood, there is evidence that they develop as a result of differences in spatial patterns of herbivore foraging [22,24,25]. It is further hypothesized that this behaviour in herbivores reflects constraints imposed by predation risk, where herbivores reduce the spatial extent of their grazing to avoid unsheltered, high predation-risk areas further from patch reef refuges [19,26,27]. Regardless of the ultimate mechanism, this pattern of spatially constrained herbivore grazing results in zones of highly grazed sandy substrate close to patch reefs, and zones of elevated algal or seagrass biomass with increasing distance from the reef (Fig 1). This foraging behaviour by herbivores, and the resulting pattern in plant biomass, has the potential to influence benthic communities through several direct and indirect mechanisms similar to those of terrestrial systems, such as a reduction in primary producer food availability and therefore benthic invertebrate abundances [7,28], with flow on effects to secondary benthic consumers [29]. It is important to note that unlike terrestrial plants that maintain complex root and rhizome structures, aquatic macroalgal beds do not have true roots, thereby limiting their below-ground biomass, and potentially, their effects on benthic invertebrate populations. However, due to both the well understood reliance of benthic invertebrates on macroalgae as a food resource [30–32], and the globally ubiquitous existence of these spatially structured herbivore grazing patterns in habitats with more complex root structures (e.g. seagrasses) [22,25,26,33], cascading effects of herbivores in grazing halo systems still have the potential to reach benthic invertebrates. To date no study has looked at how the development of these large-scale, commonly-occurring vegetation patterns affects the distribution and abundance of benthic invertebrate communities.

To assess if and how the spatial distribution of above-ground grazing affects benthic communities in a ‘natural’ aquatic ecosystem unaffected by human activities (e.g. removal of top predators), we examined the distribution and community variation in sedimentary invertebrates in a series of well-studied algal grazing halos within the lagoon of Heron Island on Australia’s Great Barrier Reef (GBR). Heron Island’s lagoonal patch reefs represent an ideal location to study the effects of herbivory on benthic communities because satellite imagery has documented the occurrence of grazing halos in the algal beds surrounding these patch reefs in the lagoon dating back to at least 1999. Additionally, Madin et al. [22] found that algal consumption by herbivores significantly decreased with increasing distance from the patch reef concomitantly with algal canopy height increases, demonstrating that herbivory is likely a key mechanism underlying the occurrence of halos in this system.

To better understand the mechanisms behind benthic invertebrate patterns, we analysed the relationship between benthic invertebrate community composition and distance from patch reef edge. We then examined the potential drivers behind the observed patterns in the benthic communities. First, we investigated whether herbivore-mediated changes in algal canopy height with increasing distance from the reef edge explain benthic community composition. We then tested the alternative hypothesis that physicochemical processes (e.g. hydrodynamics, reef erosion, etc.), unrelated to grazing, could be influencing benthic communities. For this hypothesis we focused on sediment particle size as interstitial meiofauna’s diversity and composition is known to depend highly on sedimentary conditions [34]. We also investigated the effect of carbon to nitrogen ratios (C:N) in the sediment on benthic communities, because high productivity and high fish abundance on the patch reef could cause nutrient availability to be highest adjacent to the reef [35]. This pattern, if it occurred, would not be reflective of grazing patterns, but rather would reflect nutrients transported from the patch reef. Our study presents preliminary evidence of aquatic above-ground cascading effects indirectly affecting specific taxa of benthic invertebrate communities through algal grazing, discusses the mechanisms through which these effects may be transmitted, and highlights the areas of research required to further tease apart these dynamics.

## Methods and materials

### Study area and survey design

This study was conducted in the shallow waters (< 5 m) of Heron Island lagoon (23°27′S, 151°55′E) in the southern GBR. All sampling was conducted between November and December, 2014. Heron Island is part of the Capricorn-Bunker Group, a collection of 21 coral reefs within the Mackay / Capricorn Management Area of the GBR Marine Park. Between November–December Heron Island has an average maximum temperature of 28.5°C, an average minimum temperature of 22.8°C and an average rainfall of 72.05 mm [36]. Roughly half of Heron Island’s reefs are within a no-take area of the Marine National Park Zone, while the other half lie within a limited-use area of the Conservation Park Zone, subject to strict permit issue and limited forms of recreational fishing [37]. All patch reefs in this study lay within an area of ~0.81 km^2^, located within the former zone. Benthic invertebrate samples, sediment grain size, sediment nutrient samples, and algal surveys, were taken three hours prior to, and three hours after peak high tide. For direct comparison with previous work on Heron Island’s grazing halos, satellite imagery and GPS coordinates were used to select 14 patch reef sites that were the same as those of Madin et al. [22].

At each of the 14 patch reefs, a single transect was run from the reef edge to a distance of 30 metres. The longest diameter of each patch reef was of a similar size, averaging 17.8 ± 2.3 m. Transects were placed so as to leave the maximum distance between the end of each transect and any adjacent patch reefs (72.82 ± 18.28 m). A single invertebrate core sample, a single core sample for grain size and nutrients, and three individual algal surveys were taken at each of the six distances: 0 m (just off the reef), 3 m, 7.5 m, 15 m, 22 m and 30 m (Fig 1), roughly covering an equal area along both the inside and outside of the halos. This study was carried out in accordance with the Great Barrier Reef Marine Park Act (1975) and all protocol was approved by the Great Barrier Reef Marine Park Authority (Permit numbers: G14/37304.1 & G14/37182.1). No vertebrate fauna were taken or harmed, and no endangered or protected species were involved in this study.

### Invertebrate collection and processing

Benthic invertebrates were sampled to 5 cm depth in the sediment using a 4 cm diameter corer. Surface invertebrates were not separated and as such both epibenthic and infaunal invertebrates are included in this study. Samples were preserved in sealed plastic bags with 40 ml of 70% ethyl alcohol to reduce organic matter decay. In the laboratory, benthic invertebrate sediment samples were rinsed through a 250 μm mesh sieve to separate finer particles, thereby decreasing the turbidity of the samples to facilitate increased detection of the organisms. For community abundance analyses all Polychaeta and Mollusca were classified to family level, while Nematoda and Arthropoda were identified to phylum. The diversity of each sample was calculated using the Shannon-Wiener Index [38] in conjunction with the lowest level of each taxon described above.

### Environmental data collection and processing

The canopy height of benthic primary producers, comprising of only *Enteromorpha spp*. (synonimised now under genus *Ulva*), *Cladophora spp*. and *Hincksia spp*. (a small, fine brown algae), were recorded at each distance interval by using the average of three measurements taken *in situ* with callipers (± 1 mm error). At each site and distance a second sediment core (5 cm height, 4 cm diameter) was taken to measure mean particle size and for C:N analysis. Mean particle size was calculated using a Mastersizer particle size analyser (Malvern Instruments, Malvern United Kingdom); all samples underwent a two-minute sonication pre-treatment for increased accuracy of finer particulates [39]. C:N was measured using a Costech Elemental Analyzer at the University of Hawaii at Hilo’s Marine Analytical Laboratory.

### Data analyses

Benthic community data matrices (Bray-Curtis) were square root transformed for increased homoscedasticity [40]. For multivariate analyses, algal canopy height and mean particle size were normalised using Z-score transformation to account for differences in sampling units [41]. To analyse benthic invertebrate community variation, a mixed-effects PERMANOVA was used with distance from reef and particle size included as fixed terms, and patch reef identity included as a random factor to account for the multiple samples taken along each transect. Permutations were set at 9999, and significant factors were identified through step-wise removal of nonsignificant terms. The PERMDISP (Permutational Analysis of Multivariate Dispersions) function was used to determine whether significant PERMANOVA *p*-values were a result of variance around or between means. Distance Based Linear Models (DISTLM) were then used to determine the contribution of environmental covariates algal canopy height and mean sediment particle size to the overall multivariate assemblage variation [42]. Similarity Percentages Analyses (SIMPER) using overall community abundances were used to highlight groups driving any dissimilarity between distances.

Following initial analyses of the whole data set, and based on SIMPER percent contribution, benthic invertebrate assemblages were divided into their corresponding taxa: Polychaeta, Nematoda, Mollusca and Arthropoda. Individual groups were then analysed against distance from reef, algal canopy height, mean particle size and C:N ratio using linear mixed-effects models (LME) from the “lme()” function within “nlme” package in R [43], with patch reef again treated as a random factor. Invertebrate abundance homogeneity of variance at each distance level was confirmed. A polynomial (quadratic) equation was applied to distance from patch reef to allow for curvilinear relationships. Due to co-linearity between distance from patch reef and algal canopy height, where a quadtratic distance term was found to be insignificant it was removed completely and linear models were re-run [44]. Variables that made significant contributions to taxa-specific patterns were identified through Akaike Information Criterion (AIC) and step-wise removal of nonsignificant terms. The “predictSE.lme()” function, within the “AICcmodavg” package was implemented to approximate 95% confidence intervals of model fixed effects using the delta method. Two core samples from separate patch reefs at distances 30 m and 22 m were not used in the analyses due to missing nutrient and sediment particle size data, respectively. Data were analysed using PRIMERv6 (Primer-E Ltd, Plymouth, UK), PERMANOVA+ (Permutational Multivariate Analysis of Variance) and R 3.1.0 [45].

## Results

### Environmental patterns

**Fig 2:**
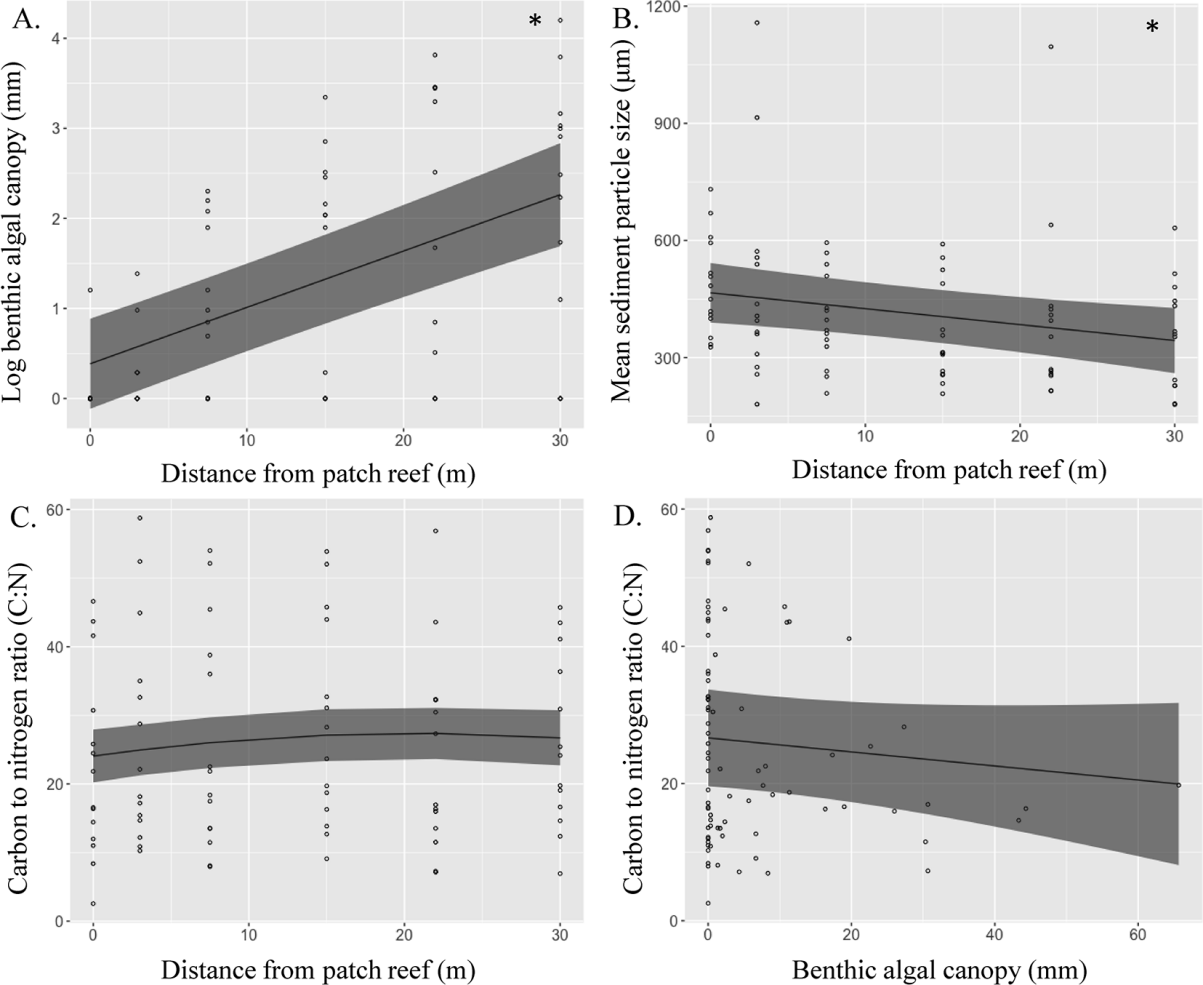
Linear mixed-effects models showing environmental patterns in the grazing halos of Heron Island lagoon. Patch reef is treated as a random factor within the model to account for between site dissimilarity. Benthic algal canopy is expressed as height (mm), * indicates significance. Solid line indicates the predicted LME model fit, and dashed lines represent 95% confidence interval; N patch reefs sampled = 14.

Algal canopy height had a significant positive relationship with distance from patch reef (LME, *f*_(1,67)_ = 76.631, *P* < 0.001, Fig 2A), and as such, these variables were not used together as predictors of benthic abundances through LMEs, and instead the best predictor was selected through the use of AIC. In contrast, mean surface sediment particle size (μm) showed a significant linear decrease with distance away from patch reef (LME, *f*_(1, 67)_ = 8.055, *P* = 0.006, Fig 2B). Sediment C:N did not significantly vary with distance from patch reef (LME, Polynomial fixed effects, *P* > 0.05, Fig 2C), and likewise were not found to be significantly associated with algal canopy height (LME, *P* > 0.05, Fig 2D).

### Invertebrate communities

A total of 4832 benthic invertebrates were identified. Overall, benthic invertebrate community composition matrices consisted of 14 groups, made up by arthropods (42%), molluscs (1.6%), nematodes (31.2%), and 11 groups of polychaetes (25.2%), the latter identified down to the lowest taxonomic level possible (i.e. family or unknown). Arthropoda consisted predominantly of harpacticoid copepods while Mollusca of bivalves. Each of these taxa were recorded as the total abundance within the sediment sample, and the multivariate analyses used taxa abundances, rather than taxa presence/absence to quantify differences among communities. Benthic communities significantly varied with distance from patch reef edge (*Pseudo-f*_(5, 65)_ = 1.489, *P* = 0.038). Pair-wise analyses (Student–Newman–Keuls) indicated significant community changes between distances 3–22 m (SNK, df, 13, *t*, 1.624, *P* = 0.022) and 3–30 m (SNK, df, 13, *t*, 1.664, *P* = 0.022), while all other comparisons between distances indicated multivariate community homogeneity (*P* > 0.05). However, marginal tests resulting from DISTLM analyses indicated that particle size (*Pseudo-f* = 1.078, *P =* 0.352) and algal canopy height (*Pseudo*-*f* = 1.368, *P* = 0.205) were not significant contributers to this overall community variation. A linear-mixed effects model was best fit to invertebrate diversity (Shannon-Weiner Index) with variables algal canopy height (LME, *f*_(1, 66)_ = 0.268, *P* = 0.606) and C:N ratios (LME, *f*_(1, 66)_ = 0.918, *P* = 0.342), showing no significant correlation with either variable.

Community composition data were found to have heterogeneous variances around group mean values (PERMDISP, *f*_(5, 78)_ = 3.48, *P* = 0.023). This variation in dispersions may have inflated the risk of type I error in community analyses, however this study retained a well-balanced experimental design, enhancing the robustness of PERMANOVA towards heterogeneity [46]. Due to 51% of benthic invertebrate sampling cores containing no molluscs and the remaining samples averaging an abundance of 1.02 ± 0.1, the observed variations in Mollusca abundances were considered too weak to interpret further through linear mixed-effects models.

**Fig 3:**
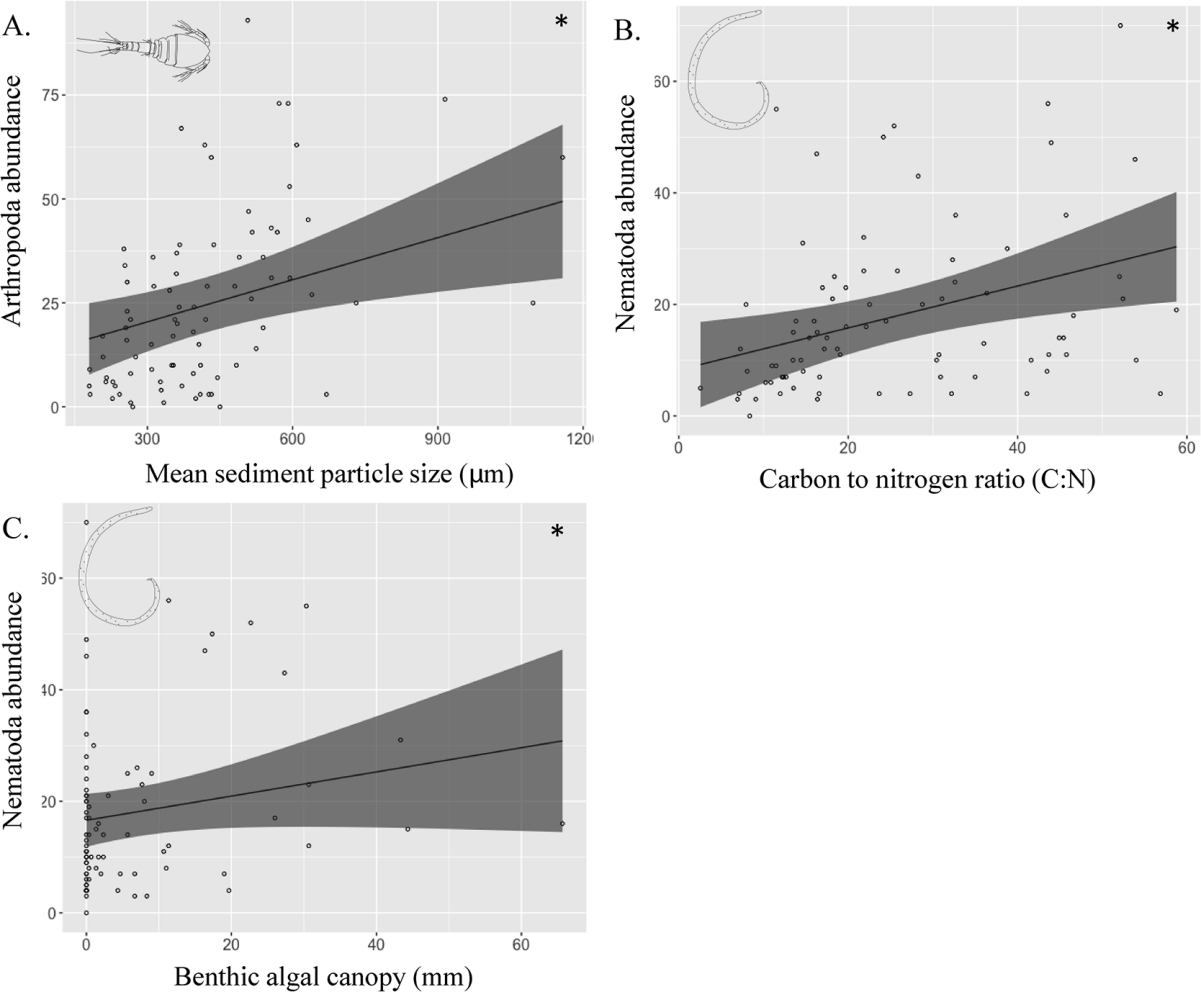
Linear mixed-effects models showing the relationships between grouped taxon abundances and environmental conditions in the grazing halos of Haron Island Lagoon. Patch reef is treated as a random factor within the model to account for between site dissimilarity. * indicates significance in the model. Solid line indicates the predicted LME model fit, and dashed lines represent 95% confidence interval; N patch reefs sampled = 14.

Taxa-specific patterns were analysed to investigate the differences in community composition with distance from the patch reef and the mechanisms behind these differences. SIMPER analysis showed the highest contributors to community dissimilarity were the abundances of Arthropoda (19.81 ± 0.48%) followed by Nematoda (14.23 ± 0.57%), and Family; Dorvilleidae (Polychaeta, 12.75 ± 0.39%), with the residual dissimilarity attributed to the remaining 12 groups of Polychaeta, and grouped Mollusca (< 10% contribution per group). Linear mixed-effects models, using grouped invertebrate abundances and AIC model selection, were used to highlight distribution patterns across grazing halos. Total invertebrate abundances were best modelled linearly with only distance from reef included, showing a nonsignificant relationship (LME, *f*_(1, 67)_ = 1.794, *P* = 0.185). Polychaeta-specific abundance were non-significantly related to both algal canopy height (LME, *f*_(1, 66)_ = 0.643, *P* = 0.4254) and mean sediment particle size (LME, *f*_(1, 66)_ = 2.137, *P* = 0.149). Arthropoda abundances exhibited a significant positive relationship with mean sediment particle size (LME, *f*_(1, 66)_ = 6.070, *P* = 0.0163, Fig 3A), but a nonsignificant relationship with algal canopy height (LME, *f*_(1, 66)_ = 3.496, *P* = 0.066). In contrast, Nematoda abundances were found to be positively related to algal canopy height (LME, *f*_(1, 66)_ = 5.149, *P* = 0.027, Fig 3C), though there was increased uncertainty in the model around higher canopy heights. In addition, Nematoda abundances were significantly greater where higher sediment C:N was present (LME, *f*_(1, 66)_ = 10.330, *P* = 0.002, Fig 3B).

## Discussion

In aquatic ecosystems, benthic community structure is fundamental to both carbon and nutrient cycling at the sediment-water interface [6,8,30,31,47]. Abiotic drivers of benthic communities have been extensively studied [48–51], however little is known regarding the effects of above-ground biotic processes, and their possible cascading influence on benthic biota. This study of grazing halos in Heron Island lagoon demonstrates that the major factors influencing spatial differences in benthic invertebrates are taxon-dependent. While Nematoda responded significantly to both spatial differences in algal canopy height and sediment nutrient concentrations, Athropoda responded only to sediment particle size and Polychaeta was unaffected by any of our measured variables.

The only benthic taxon that showed a significant relationship with benthic algae was Nematoda, whose abundances were positively related to algal canopy height. This relationship between algae and Nematoda abundance is likely related to the fact that omnivorous nematodes, which have the ability to manipulate and digest large algal particles, have been shown to make up a large portion of the total Nematoda populations on the reef shelves and lagoons of the GBR [52]. In addition, marine nematodes are known to display spatial aggregations in their abundances [53], which have been suggested to be a result of selective consumption and settlement in optimal areas [54–56]. For example, experiments by Ullberg and Ólafsson [56] demonstrated that Nematoda suspended in the water column are able to choose settling areas, displaying a clear preference for sediments with benthic algae, where their abundances were up to seven times higher than those of bare substrates.

In addition, several physicochemical properties of the sediments can also influence benthic communities. For example, variation in sediment particle size, largely attributed to physical weathering of coral structures and hydrological regimes [57,58], is widely accepted as a major driver of benthic invertebrate abundances in aquatic ecosystems [59–62]. Sediment particle size shapes benthic biota by creating conditions in which optimal body sizes are required for both interstitial living and sediment reworking [59], while also affecting sediment water and nutrient concentration [63], pore water oxygen exchange, and redox environment [64]. All are fundamental to benthic community structure [65]. In accordance with our hypothesis and previous literature, mean sediment particle size exerted a positive influence on Arthropoda abundances (predominantly copepods).

Sediment nutrient content (C:N) displayed a taxon-specific positive relationship with benthic communities, specifically Nematoda abundances. One mechanism that can alter nutrient levels in surface sediments is fish faecal deposition and the transportation of nutrients from the reef [35,62,66]. As fish reduce their foraging distance, they simultaneously spend an increased amount of time closer to their patch reef refugia [67], potentially resulting in higher nutrient concentrations in these areas. Sediment carbon content not only affects the amount of easily broken down organic matter directly available for benthic invertebrate consumption, but also promotes differering communities of bacteria (C:N >30:1) and fungi (C:N <30:1), with flow on effects to bactivorous and fungivorous invertebrates [68]. However, C:N ratios in the sediments were not significantly related to distance from patch reef or algal canopy height. These results suggest that the drivers of sediment nutrient content, independent from fish faecal deposition and algal growth, also play a key role in shaping benthic invertebrate abundances in these systems.

Separate from the cascading effects of herbivores through primary producer distribution, and outside the scope of this study, are the consumptive effects of fish predation on benthic invertebrate abundances [13]. Invertivorous coral reef fish species are theoretically subject to the same behavioural pressures as those of their herbivorous neighbours, and as such would potentially exert direct effects on benthic invertebrate abundances along a distance from patch reef gradient. These direct effects would likely have a strong influence on the benthic invertebrate distributions analysed here. Under this scenario, distance from patch reef could be used as a proxy for invertivorous fish foraging. However, within this study linear mixed effects models on grouped invertebrate abundances, with AIC model selection including all covariates, indicated that distance from patch reef should be excluded for greater model fit (apart from total abundances that showed a nonsignificant relationship with distance). As such, we can assume that the relationships between algal canopy height and benthic invertebrates observed here are robust to predatory effects.

The primary goal of this study was to investigate potential cascading influences of above-ground aquatic grazing, through changes to benthic primary production, on benthic invertebrate communities. Our findings suggest that only Nematoda populations responded to differences in benthic algal canopy height indicating that although present, cascading effects of this type are a weak driver of benthic communities in this benthic algal dominated system. We also found that the observed cascading effects exert influence on benthic communities in conjunction with other well known environmental factors (i.e. sediment particle size and sediment C:N). These results suggest that in this system, benthic invertebrate communities and the ecosystem functions they perform, may be less responsive to changes in above-ground processes than those that have been studied in terrestrial systems. Further studies are needed to understand whether the observed benthic invertebrate community response to above-ground herbivory found in this study is ubiquitous across both temporal scales (i.e. fluctuations in algal biomass) and other marine vegetated ecosystems.

## Supporting information

S1 File Invertebrate abundances with distance away from patch coral reef. Excel file that consists of algal canopy height (mm), grouped invertebrate abundances, mean sediment particle size (μm), carbon to nitrogen ratios (C:N) and diversity (Shannon-Weiner index) at each distance interval away from patch reef (m); 0, 3, 7.5, 15, 22 and 30.
(XLSX)
